# Comparative myoanatomy of Tardigrada: new insights from the heterotardigrades *Actinarctus doryphorus* (Tanarctidae) and *Echiniscoides sigismundi* (Echiniscoididae)

**DOI:** 10.1186/s12862-019-1527-8

**Published:** 2019-11-06

**Authors:** Dennis Krog Persson, Kenneth Agerlin Halberg, Ricardo Cardoso Neves, Aslak Jørgensen, Reinhardt Møbjerg Kristensen, Nadja Møbjerg

**Affiliations:** 1 Natural History Museum of Denmark, Department of Biology, University of Copenhagen, Universitetsparken 15, DK-2100 Copenhagen Ø, Denmark; 2 Present Address: Department of Chemistry and Molecular Biology, University of Gothenburg, Medicinaregatan 9C, 405 30 Gothenburg, Sweden; 3 Section for Cell- & Neurobiology, Department of Biology, University of Copenhagen, Universitetsparken 15, DK-2100 Copenhagen Ø, Denmark; 4 Department of Biology, University of Copenhagen, August Krogh Building, Universitetsparken 13, DK-2100 Copenhagen Ø, Denmark

**Keywords:** Tardigrada, Myoanatomy, Phalloidin, Phylogeny, Ecdysozoa

## Abstract

**Background:**

Tardigrada is a group of microscopic invertebrates distributed worldwide in permanent and temporal aquatic habitats. Famous for their extreme stress tolerance, tardigrades are also of interest due to their close relationship with Arthropoda and Cycloneuralia. Despite recent efforts in analyzing the musculature of a number of tardigrade species, data on the class Heterotardigrada remain scarce. Aiming to expand the current morphological framework, and to promote the use of muscular body plans in elucidating tardigrade phylogeny, the myoanatomy of two heterotardigrades, *Actinarctus doryphorus* and *Echiniscoides sigismundi*, was analyzed by cytochemistry, scanning electron and confocal laser scanning microscopy and 3D imaging. We discuss our findings with reference to other tardigrades and internal phylogenetic relationships of the phylum.

**Results:**

We focus our analyses on the somatic musculature, which in tardigrades includes muscle groups spanning dorsal, ventral, and lateral body regions, with the legs being musculated by fibers belonging to all three groups. A pronounced reduction of the trunk musculature is seen in the dorsoventrally compressed *A. doryphorus*, a species that generally has fewer cuticle attachment sites as compared to *E. sigismundi* and members of the class Eutardigrada. Interestingly, F-actin positive signals were found in the head appendages of *A. doryphorus*. Our analyses further indicate that cross-striation is a feature common to the somatic muscles of heterotardigrades and that *E. sigismundi—*as previously proposed for other echiniscoidean heterotardigrades—has relatively thick somatic muscle fibers.

**Conclusions:**

We provide new insights into the myoanatomical differences that characterize distinct evolutionary lineages within Tardigrada, highlighting characters that potentially can be informative in future phylogenetic analyses. We focus our current analyses on the ventral trunk musculature. Our observations suggest that seven paired ventromedian attachment sites anchoring a large number of muscles can be regarded as part of the ground pattern of Tardigrada and that fusion and reduction of cuticular attachment sites is a derived condition. Specifically, the pattern of these sites differs in particular details between tardigrade taxa. In the future, a deeper understanding of the tardigrade myoanatomical ground pattern will require more investigations in order to include all major tardigrade lineages.

## Background

The phylum Tardigrada consists of over 1200 species of microscopic invertebrates, which are famous for their extreme stress tolerance [1–4]. Tardigrades have a worldwide distribution, inhabiting marine, freshwater and terrestrial habitats [5, 6]. Apart from their impressive stress tolerance, tardigrades are also of great interest due to the widely accepted scenario of a close phylogenetic relationship with Arthropoda (e.g., insects, crustaceans) and Onychophora (velvet worms), within Ecdysozoa (moulting animals; for a review see [7]). Tardigrades have, however, also been recovered as sister group to Nematoda (e.g., *C. elegans*) and their exact phylogenetic affinities thus remain controversial [7, 8]. The internal phylogeny of Tardigrada is also, from a morphological stand point, often difficult to resolve due to the relative scarcity of useful characters.

Extant tardigrades are represented by two major evolutionary lineages, the well investigated Eutardigrada (including the newly erected Apotardigrada) and the lesser known and much more diverse Heterotardigrada [9–13]. The taxonomy and internal phylogeny of tardigrades was traditionally based on adult morphological features, such as cuticular structures (e.g., claws, sensory organs, pharyngeal placoids, and cuticular ornamentation) as well as egg morphology (e.g. [14–21]). However, studies addressing tardigrade phylogeny have shifted more towards the use of DNA sequencing, which has had a great impact on this research field (e.g. [22, 23]). Importantly, the addition of molecular techniques has changed the internal phylogeny of Heterotardigrada by including Echiniscoidea within Arthrotardigrada, making the latter order paraphyletic [9, 11, 22–24]. It has become increasingly apparent that in order to advance towards a modern classification, it is essential to combine both morphological/anatomical and molecular data. Indeed, it has been suggested that the use of neuro- and myoanatomical structures can be useful for establishing a reliable taxonomic hierarchy and resolving the internal phylogeny of Tardigrada [25–32].

In order to use myoanatomical variations as informative characters to assess phylogenetic relationships, the amount of data on the diversity of tardigrade myoanatomy must be significantly increased. This is especially true for Heterotardigrada, the class within Tardigrada which exhibits the highest variation in external morphology, and likely also possesses useful myoanatomical variation [12, 25–32]. Moreover, as heterotardigrades are considered to possess many ancestral tardigrade characters, an understanding of the myoanatomy and its diversity within Heterotardigrada will help us understand the evolutionary history of tardigrades. Here, we use phalloidin staining, confocal laser scanning microscopy (CLSM) and 3D imaging to provide a detailed description of the myoanatomy of two heterotardigrades, namely *Actinarctus doryphorus* (Tanarctidae) and *Echiniscoides sigismundi* (Echiniscoididae). In addition, we give a short overview of the external morphology of the two species using scanning electron microscopy (SEM). Investigations of tardigrade anatomy date back to the nineteenth century [12] and we note that Marcus [33] and Müller [34]—based on light microscopic investigations—provided drawings of the musculature in *Echiniscoides sigismundi* [33] as well as *Echiniscus testudo*, *Milnesium tardigradum*, *Ramazzottius oberhaeuseri* and *Macrobiotus hufelandi* [34]. Importantly, these early studies have provided a thorough basis for present day investigations. Here, we compare our results with contemporary myoanatomical data obtained from different tardigrade taxa and discuss potential homologies of character states.

## Results

Tardigrades are bilaterally symmetric and the following descriptions thus apply solely to one side of the body unless otherwise stated. The description below follows the nomenclature used by Schmidt-Rhaesa and Kulessa [30], Halberg et al. [35], Schulze and Schmidt-Rhaesa [31], Halberg et al. [25] and Smith and Jockusch [32]. Muscle names are, when possible, derived from an attachment site and the muscle group that the respective muscle is part of. As an example, ventral leg muscles, *i* and *iii*, which connect to ventromedian attachment sites, are denoted with either *i* or *iii* preceded by a number that indicates which of the seven attachment sites the specific fiber is associated. For example, *1i* or *2i*, indicate leg muscle *i* from ventromedian attachment sites *1* and *2*, respectively. In case more muscle fibers from one group originate from the same attachment site, numbers will be added sequentially after a hyphen at the end of the muscle name, e.g., *1i-1*, *1i-2* and so on.

### Gross morphology of *Actinarctus doryphorus* (Fig. 1)

**Fig. 1:**
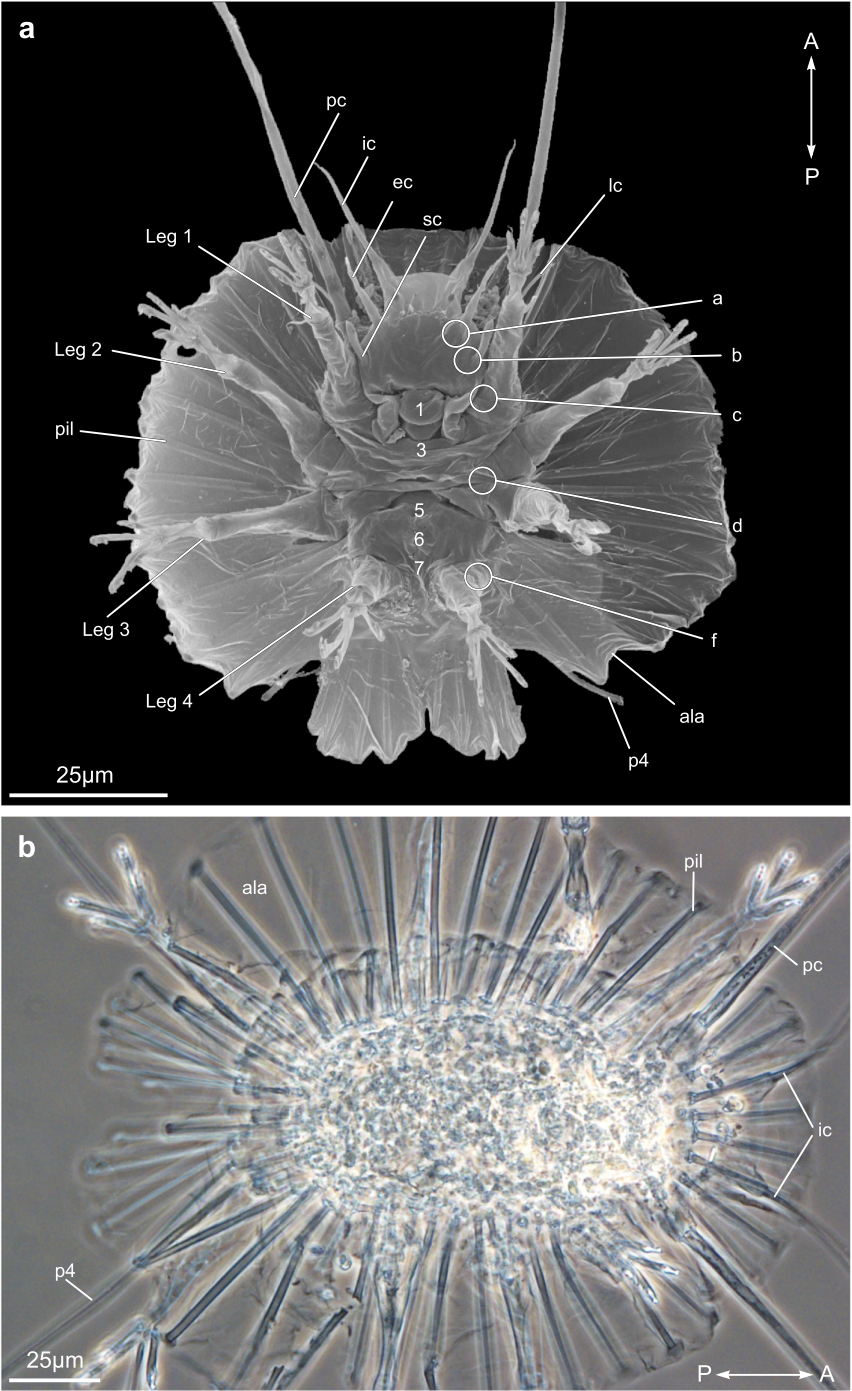
Habitus of *Actinarctus* cf. *doryphorus.* **a** Scanning electron microscope image showing the ventral side. **b** Light microscope image showing the dorsal side. Abbreviations: 1–7, approximate position of ventromedian attachment sites; a-f, approximate position of intermediate ventral attachment sites; ala, wing-like extension of the cuticle; ec, external cirri; ic, internal cirri; lc, lateral cirri; pc, primary clavae; pil, pillar; p4, sensory organ of the fourth leg; sc, secondary clavae. Double arrow indicates the anterior (A) -posterior (P) direction

*Actinarctus doryphorus* has a dorsoventrally compressed body with a length of 150–175 μm [36]. Notably, the dorsal cuticle forms a conspicuous wing-like structure (Fig. 1; *ala*), which is supported by epicuticular pillars (Fig. 1; *pil*). When viewed from the ventral side the distinct head region is clearly visible (Fig. 1a). The head appears relatively large and it is equipped with five sets of sensory appendages: primary clavae (Fig. 1; *pc*), secondary clavae (Fig. 1; *sc*), lateral cirri (Fig. 1; *lc*), external cirri (Fig. 1; *ec*) and internal cirri (Fig. 1; *ic*). The primary clavae are very long, sometimes exceeding the length of the body. In addition, the head region bears a single dorsal median cirrus (not shown) only visible from the dorsal side. Posteriorly the trunk bears a dorsolateral cirrus on each side (not shown). The four pairs of legs are telescopic and terminate in four toes, each bearing a retractable claw (Fig. 1). Additionally, each leg bears a sensory organ. The sensory organ of the fourth leg (*p4*) penetrates the ala and extends onto the dorsal surface (Fig. 1) [37].

### Myoanatomy of *Actinarctus doryphorus* (Figs. 1-2 )

**Fig. 2:**
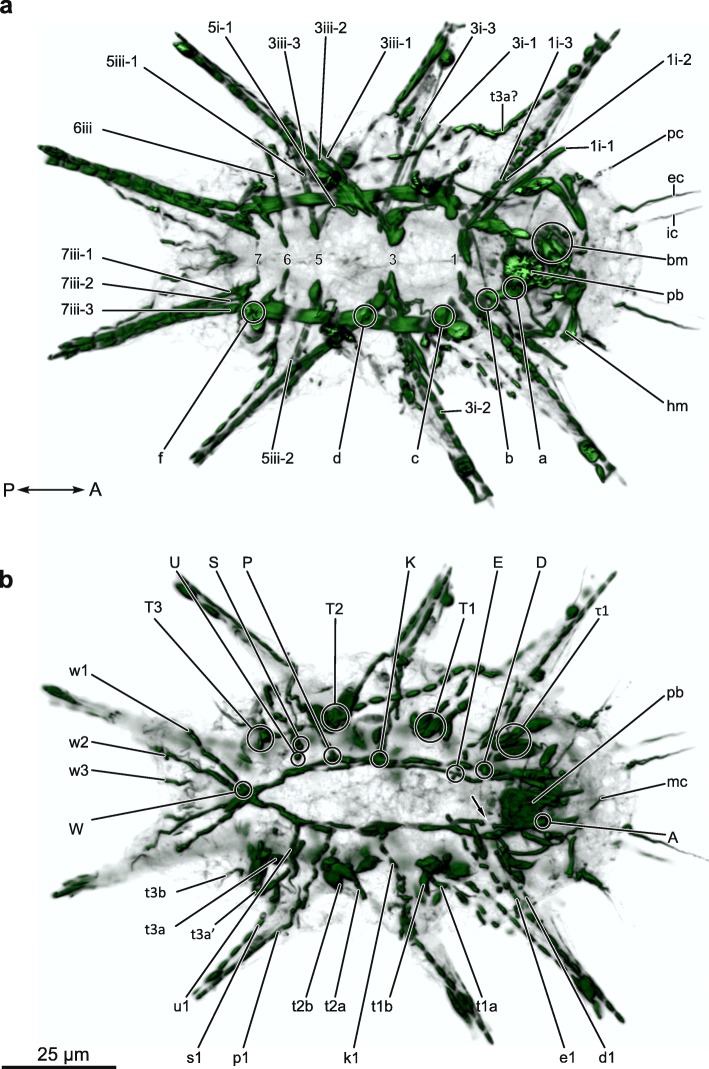
3D reconstruction of the musculature of *Actinarctus doryphorus*. **a** ventral and **b** dorsal view of the same specimen. Abbreviations: 1–7: ventromedian attachment site; a-f: intermediate ventral attachment sites; A-W, dorsal attachment sites (associated muscles: corresponding lowercase letter followed by a number); bm, buccal muscles; ec, external cirri; hm, head muscles; ic, internal cirri; mc, median cirri; pc, primary clavae; sc, secondary clavae

The phalloidin staining of the somatic musculature in most of our *A. doryphorus* specimens revealed a clear truncated signal, indicating a cross-striated musculature (Fig. 2 and Additional file 1). The following description of the somatic muscles in *A. doryphorus* is focused on the musculature of the legs, the dorsal and ventral musculature, including muscles of the head region. While a reduced number of lateral attachment sites can be recognized, the lateral muscles appear reduced and are not easily discernable as a distinct muscle group in our F-actin stainings.

#### Ventromedian attachment sites of the trunk and leg muscles

Five paired ventromedian attachment points were recognized along the anterior-posterior body axis by CLSM analyses of phalloidin stained specimens (Fig. 2a). Their position within the trunk, relative to the four leg pairs, indicate that these anchoring points correspond to ventromedian attachment *sites* *1*, *3*, *5*, *6* and *7* as described from eutardigrades (Fig. 1) [12, 25, 35]. Thus *A. doryphorus* seems to have lost attachment *sites 2* and *4*. The ventromedian attachment sites of *A. doryphorus* were not clearly visible as depressions in the cuticle of SEM preparations as reported from other tardigrades. Therefore, their positions, as indicated on Fig. 1a, are only approximate. The five paired ventromedian attachment points form a metameric pattern, each with a unique architecture determined by differences in the attachment of the ventral leg musculature (Fig. 2a). Specifically, ventral leg muscles (*i* and *iii*) connect with the attachment points. Our analysis failed to reveal any ventrolateral muscles (labeled “*ii*” in *Echiniscoides sigismundi*; see below), spanning the body from ventromedian to lateral attachment points.

At the most anterior ventromedian attachment site (*site 1*), muscle *1i-2* extends into the distal part, whereas the muscle *1i-3* connects with the proximal part of the first leg (Fig. 2a). Muscle *1i-1* extends anteriorly with a termination point in the proximal part of the first leg or in the head region. The muscles *3i-1* and *3i-3* span from the proximal region of the second leg to attachment *site 3*, whereas muscle *3i-2* attaches distally in the second leg (Fig. 2a). The musculature of the third leg is composed of muscles *3iii-1*, *3iii-2*, *3iii-3*, *5i-1*, *5iii-1*, *5iii-2* and *6iii* (Fig. 2a). The muscles *3iii-2* and *3iii-3* extend from attachment *site 3* into the distal part of the leg (Fig. 2a). Also, from attachment *site 3*, the muscle *3iii-1* connects to the proximal part of the third leg. Muscle *5iii-2* extends from attachment *site 5* to the distal part of the third leg, whereas muscle *5i-1*, *5iii-1* and *6iii* extend to the proximal part of the third leg from *sites 5* and *6*, respectively (Fig. 2a, Additional file 1). The musculature of the hind leg is composed of the muscles *7iii-1*, *7iii-2* extending from the most posterior ventromedian attachment site (*site 7*). In addition, the muscle *7iii-3* extends into the fourth leg connecting to the thick ventral longitudinal musculature at intermediate attachment site *f* (Fig. 2a). There seems to be a pattern with regard to the number of ventromedian attachment sites that each of the first three legs are associated with: the ventral muscles of the first leg are all attached to ventromedian attachment site *1*, while ventral muscles of the second leg pair attach to ventromedian attachment *site 3* and *5*, and ventral muscles of the third leg pair attach to attachment site *3*, *5* and *6* (Fig. 2a). The ventral muscle fibers of the hind legs are attached only to attachment site *7* and to the ventral longitudinal musculature (Fig. 2a)

In addition, muscles of the legs are also found associated with dorsal attachment sites (below follows a description of the dorsal musculature). Specifically, the musculature of the first leg includes the dorsally connected muscles *e1* and *d1*. Muscle *k1* connects to the second leg, whereas *p1*, *s1, u1* and *w1, w2*, *w3* extend into, respectively, the third and fourth leg pairs (Fig. 2b).

The musculature of the legs furthermore comprises fibers pertaining to a much reduced lateral muscle group. The musculature of the first leg includes the lateral muscle *t1a* connecting to lateral attachment site *T1* (Fig. 2b). The lateral muscle *t2a* extends from lateral attachment site *T2* and into the second leg (Fig. 2b). From lateral attachment site *T3*, in the most posterior region of the animal, the lateral muscle *t3a* may extend all the way into the first leg (Fig. 2a, b), we however state this with caution as we have been unable to confirm the course of this muscle in other specimens. The muscle *t3a’* seems to connect with the proximal part of the third leg (Fig. 2b) and the muscle *t3b* seems to connect *T3* to the proximal region of the hind leg (Fig. 2b, Additional file 1).

#### Dorsal longitudinal musculature

Two dorsal longitudinal muscles extend in parallel along the anterior-posterior body axis (Fig. 2b). The two strands, the outer and inner muscle strand, span from anterior attachment site *A*, to posterior attachment site *W* (Fig. 2b). However, between attachment site *U* and *W* it seems that the inner and outer strand has fused into a single muscle. Compared to the ventral longitudinal muscles (see below), the dorsal longitudinal muscles are very thin and single sarcomeres cannot be clearly discerned. All of the dorsal attachment sites seem to be connected to muscles from the legs (Fig. 2b; *d*-*w*), except for the anterior most attachment point *A*. Close to attachment site *D*, we find what seems to be a discontinuity in the inner longitudinal strand (Fig. 2b, arrow).

#### Ventral longitudinal musculature and musculature of the head region

The ventral longitudinal musculature comprises a long pair of muscles within the trunk as well as two shorter pairs that pertain to the head region. The thick long pair spans the trunk from intermediate attachment site ‘*c*’—near the first leg pair—through intermediate attachment site ‘*d*’ between the second and third leg, to intermediate attachment site ‘*f*’, which is located at the fourth leg pair (Fig. 2a). Each of the long thick ventral longitudinal muscles seems to be composed of only four large sarcomeres (Fig. 2a).

The two short muscle pairs of the ventral musculature are positioned anteroventrally in the head region. One of these pairs forms a relatively thin inner band, which extends between intermediate attachment sites ‘*b*’ and ‘*a*’ and continues anteriorly along each side of the ovoid, muscular pharyngeal bulb (Fig. 2a; *pb*) in direction of muscles that seem to be associated with the stylet and mouth cone. The latter are collectively termed buccal muscles (Fig. 2a; *bm*) as our stainings do not allow for a more detailed investigation of these delicate structures. The inner band seems to be composed of two sarcomeres (see Additional file 1). In addition to this inner band, a pair of large muscles is found on each side of the head (Fig. 2a; *hm*), spanning in medial direction from a ventrolateral position. Specifically, each of these muscles is comprised of three large sarcomeres with a size comparable to the sarcomeres of the long ventral longitudinal muscles of the trunk. The first sarcomere of this thick outer band is aligned with the thick ventral longitudinal muscles of the trunk, and it continuous in an anterior direction from a ventrolateral position in front of the first leg closely associated with the secondary clavae. The second and third sarcomere of this conspicuous three sarcomere structure forms a “V” with the second sarcomere extending in medial direction towards the more anterior part of the above-mentioned buccal muscles (Fig. 2a; Additional file 1). Here the second sarcomere connects to the third sarcomere, which protrudes back towards the lateral surface and connects to fine dorsally extending muscles that associate with the proximal part of the primary clava (Fig. 2a; *pc*). The thickness of the individual sarcomeres in this outer ventral band of the head region, together with the ventrolateral position of the first sarcomere, indicates that this structure derives from the ventral trunk muscle. Notably, phalloidin positive signals are found inside the proximal part of all head appendages (Fig. 2a, b).

### Gross morphology of *Echiniscoides sigismundi* (Fig. 3)

**Fig. 3:**
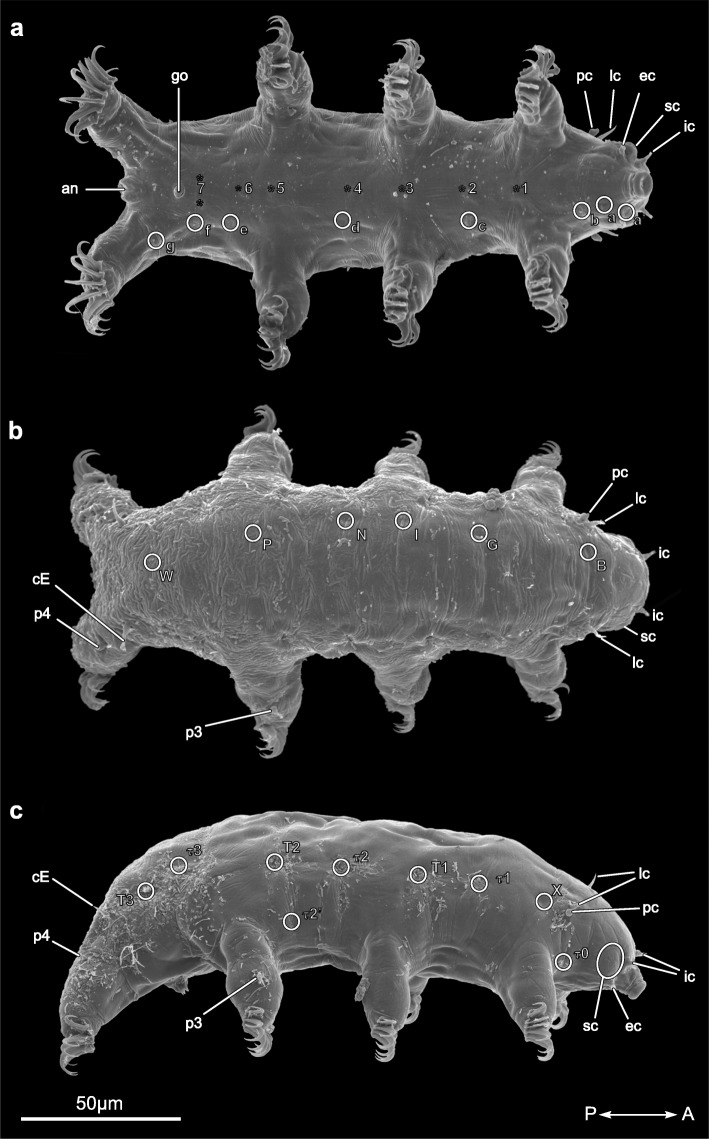
Habitus of *Echiniscoides sigismundi*. **a-c** Scanning electron microscope images. **a** Ventral side. **b** Dorsal side. **c** Lateral side. Abbreviations: 1–7, ventromedian attachment sites; a’-g, intermediate ventral attachment sites; B-W, dorsal attachment sites; T1-T3, τ0- τ3, lateral attachment sites; an, anus; go, gonopore; cE, cirrus E; ec, external cirrus; lc, lateral cirrus; ic, internal cirrus; pc, primary clavae; sc, secondary clavae; p3 + p4, sensory structures of legs. Double arrow indicates the anterior (A)-posterior (P) direction. The position given for all attachment sites are approximate

The external morphology of *Echiniscoides sigismundi* [38] is very different from that of *A. doryphorus*. The body of *E. sigismundi* is cylindrical and the legs are not telescopic. In most cases, the cuticular attachment sites are visible externally as small depressions on the surface of SEM prepared specimens; the approximate position of the remaining attachment sites are similarly indicated (Fig. 3). This echiniscoidean tardigrade is characterized by relatively short sensory appendages, which include primary clavae (*pc*), secondary clavae (*sc*) (papilla cephalica), lateral cirri (*lc*), external cirri (*ec*) and internal cirri (*ic*), as well as cirri E (*cE*) of the trunk and leg sensory appendages (*p3*, *p4*) (Fig. 3). External evidence of the heads median cirrus is absent.

### The myoanatomy of *Echiniscoides sigismundi* (Figs. 3 and 4)

#### Ventromedian attachment sites, ventral leg muscles and ventrolateral muscles (Figs. 3a and 4a)

**Fig. 4:**
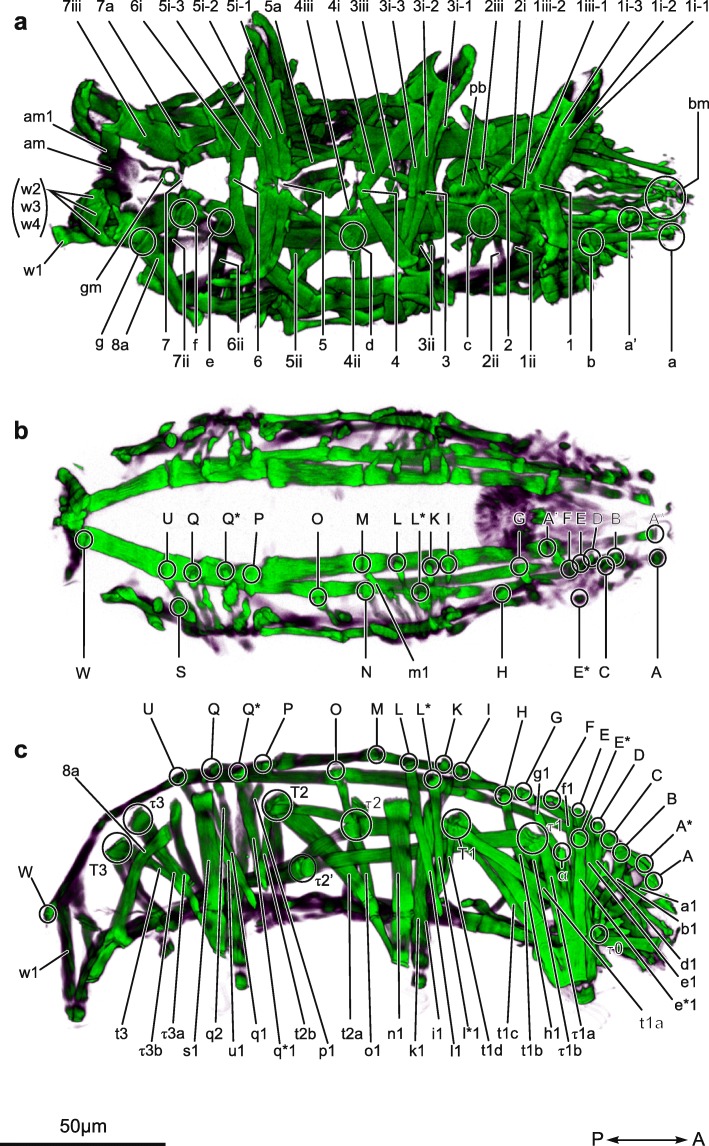
3D reconstruction of the musculature of *Echiniscoides sigismundi*. **a** ventral side. **b** dorsal side. **c** lateral side. Abbreviations: 1–7, ventromedian attachment sites (associated muscles: i and iii); a-f, intermediate ventral attachment sites (associated muscles: ii); A-W, dorsal attachment sites (associated muscles: corresponding lowercase letter followed by a number); T1-T3, τ0-τ3, lateral attachment sites; bm, buccal muscles; hm, head muscles; am, anal muscles; gm, gonopore muscle pb, pharyngeal bulb

*Echiniscoides sigismundi* possesses seven ventromedian attachment sites along the anterior-posterior body axis (Figs. 3a and 4a; *sites 1*–7), to which leg muscles (*i* and *iii*) and ventrolateral muscles (*ii*) attach. Leg muscles *1i-1*, *1i-2*, *1i-3*, *1iii-1, 1iii-2* as well as the ventrolateral muscle *1ii* connect to the most anterior *site 1* (Fig. 4a). Muscles *2i* and *2iii* converge and connect to the second ventral attachment site, as does the ventrolateral muscle *2ii* (Fig. 4a). Muscle *2i* extends into the proximal part of the first leg pair, whereas muscle *2iii* extends into the proximal part of the second leg pair. The third ventromedian attachment site serves as anchoring point for muscles *3i-1*, *3i-2* and *3i-3* from the second leg as well as the ventrolateral muscle *3ii*, and is connected to the ventral longitudinal musculature via muscle *3iii* (Fig. 4a). The muscle *4i* extends from the fourth ventromedian attachment site to the proximal part of the second leg, while the muscle *4iii* extends to the proximal part of the third leg. The ventrolateral muscle *4ii* is also connected to the fourth ventromedian attachment site. The muscle *5i-1* extends from the fifth ventromedian attachment site to the proximal part of the third leg. In addition, muscles *5i-2* and *5i-3* extend from the fifth ventromedian attachment site to the distal part of the third leg (Fig. 4a). The ventrolateral muscle *5ii* is connected also with the fifth ventromedian attachment site. Two muscles, *6i* and *6ii*, were found in association with the sixth ventromedian attachment site. The muscle 6*i* extends into the third leg, whereas 6*ii* extends laterally. The muscles *7ii* and *7iii* extend from the seventh ventromedian attachment site to the lateral side and the fourth leg, respectively. The musculature of the fourth leg pair includes one ventral muscle, *7iii* that emerges from the seventh ventromedian attachment site.

#### Ventral longitudinal musculature

The ventral longitudinal musculature consists of a single pair of muscles that span from anterior attachment sites *a* and *a’* to the posterior attachment site *g* (Fig. 4a). These muscles are composed of several fibers, which anchor at various attachment sites, namely *b*, *c*, *d*, *e* and *f*, where they connect with other muscles such as *1iii-1*, *3iii*, *5a* and *7a*. From attachment site *c* the ventral longitudinal muscle bifurcates into two muscle fibers, which extend into the anterior attachment sites *a* and *a’*, respectively (Fig. 4a).

#### Dorsal and lateral longitudinal musculature (Figs. 4b, c)

Two pairs of dorsal longitudinal muscles are present in *E. sigismundi*, a short inner pair and a long outer pair. The short inner pair extends between dorsal attachment sites A* and *P* (Fig. 4b), while the long outer pair extends between attachment sites *A* and *W* (Fig. 4b). Furthermore, the long outer pair of dorsal longitudinal muscles bifurcates at approximately one third of its length (from the anterior end), and fuses again between attachment sites *E* and *D* (Fig. 4b).

The lateral muscles of *E. sigismundi* extend longitudinally and diagonally from attachment sites *τ0* to *T1* then to *τ2* where it bifurcates to *T2* and *τ2’*, and from *τ1* to *τ2’* (Fig. 4c). Muscles from *T2* connect with the second and third leg, respectively, while a muscle from *τ2’* connects to the ventral musculature. The lateral attachment sites *τ3* and *T3* did not seem to be connected to the rest of the lateral muscles and attachment sites (Fig. 4c).

#### Dorsal and lateral leg muscles (Fig. 4c)

The first leg musculature is supplied with a total of 12 identifiable muscles from dorsal attachment sites as well as from the lateral attachment sites *τ1* and *T1*. The dorsal leg muscles *b1*, *d1*, e1, e*1, *f1*, *g1* and *h1* emerge from attachment points B, D, E, E*, F, G and H, respectively, associated with the long outer dorsal longitudinal muscle and extend into the anterior region of the leg, though it is not possible to distinguish their exact arrangement (Fig. 4c). Likewise, it is not possible to determine exactly where muscle *a1* terminates and the path of *c1* is also not discernable. The dorsal leg muscle *h1* extends from the long outer dorsal longitudinal muscle and connects to the posterior region of the leg, fusing with muscles *t1b*, *t1c*, and *τ1b* from the lateral musculature, forming a sheet-like muscle in the distal part of the posterior region of the leg (Fig. 4c). In addition, muscle *α* fuses with muscles *τ1a* and e*1 and becomes part of the sheet-like muscle in the distal part of the first leg.

A total of nine muscles with dorsal and lateral attachment were identified for the second leg. Four of these muscles, *i1*, *k1*, *l1* and *m1*, originate from the inner dorsal longitudinal muscle and three other, namely l*1, *n1* and *o1*, from the outer dorsal longitudinal muscle (Fig. 4b, c). The second leg also receives muscles *t1d* and *t2a* from their respective attachment sites (Fig. 4c). Muscle *t2a* fuses into a distal sheet-like muscle.

In the third leg five muscles, namely *p1*, *q1*, *q2,* and *u1*, originate from the outer dorsal longitudinal muscle. The muscles *p1* and *q2* connect to the distal part of the leg, whereas q*1, *q1* and *u1* extend into the proximal part of the leg (Fig. 4a). The muscles *t2b*, *τ3a* and *t3* also extend into the proximal part of the leg, whereas muscles *τ3b* and *s1* connect and form a sheet-like muscle in the distal part of the leg (Fig. 4c).

The hind legs receive four dorsal muscles, *w1, w2*, *w3* and *w4*, from attachment site *W*, which form a sheet-like muscle somewhat similar to the sheet-like muscles found in the other legs (Fig. 4a, c).

#### Musculature of the head region and gonopore

Muscles of the head include what we interpret as buccal muscles (Fig. 4a; *bm*). These muscles extend from the anteriormost region of the head towards the pharyngeal bulb (Fig. 4a). The pharyngeal bulb is located dorsally to the second ventromedian attachment site (Fig. 4a; *pb*). Musculature of the gonopore appears as a circular structure located near the seventh ventromedian attachment site (Fig. 4a; *gm*), whereas the musculature of the anus is located between the hind legs (Fig. 4a; *am*).

## Discussion

The tardigrade somatic musculature can be divided into dorsal, lateral, and ventral muscle groups with the four leg pairs receiving muscles from all three groups [12, 25, 26, 30–32, 35]. *Actinarctus doryphorus* and *Echiniscoides sigismundi* share a common myoanatomical ground pattern with other tardigrades investigated so far. However, we also found significant differences in the more detailed architecture of the two species. A number of cuticular attachment sites pertaining to all three somatic muscle groups seem to have been lost in *A. doryphorus* and the musculature of the trunk generally appears reduced and simplified in this species as compared to other tardigrades, including other heterotardigrades. Our comparative analysis indicates that this reduction represents an apomorphic condition. In *E. sigismundi*, on the other hand, the somatic musculature seems to be composed of relatively thick muscle fibers—a feature that appears to be common to echiniscoidean heterotardigrades. Below we discuss the distinct musculature of the two anatomically very diverse heterotardigrades with reference to other tardigrades and internal phylogenetic relationships of Tardigrada.

Interestingly, the somatic musculature of *A. doryphorus* is composed of sarcomeres that in our fluorophore coupled phalloidin based stains show a clear striation pattern. Cross-striation is also seen in transmission electron microscopy of *A. doryphorus*, clearly revealing the presence of Z-lines consisting of small Z-discs within leg muscles (Fig. 2.9a in ref. [12]). Specifically, these Z-discs mark the boundaries between adjacent sarcomeres, providing an anchoring point for the phalloidin stained actin filaments. A similar striation pattern is seen in the heterotardigrade *Batillipes pennaki* as well [31, 39]. Our phalloidin stains also reveal gaps in the staining between sarcomeres in the musculature of *E. sigismundi* (see Additional file 2, Additional file 3 and Additional file 4), seemingly indicating that cross-striated somatic muscles is a feature shared by heterotardigrades. The somatic musculature of eutardigrades has been described as an intermediate condition between smooth and obliquely striated [40], whereas the musculature of the pharyngeal bulb, stylets and legs seem to be cross-striated in both heterotardigrades and eutardigrades [39, 41].

**Fig. 5:**
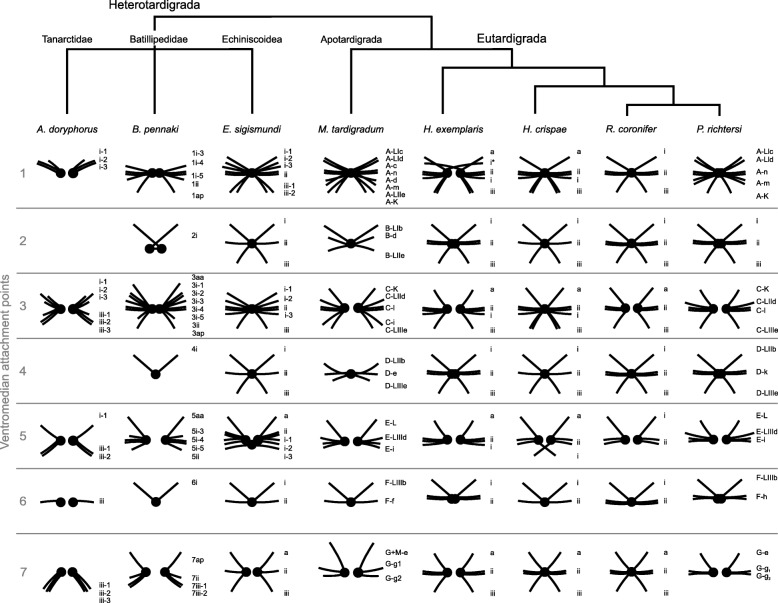
Schematic comparison of the ventromedian attachment sites and their associated muscles in tardigrades. Representation of *Actinarctus doryphorus* (this study), *Echiniscoides sigismundi* (this study), *Batillipes pennaki* [31], *Milnesium tardigradum* [30], *Hypsibius exemplaris* [32], *Halobiotus crispae* [35], *Richtersius coronifer* [25] and *Paramacrobiotus richtersi* [26] with tree topology and taxa names following recent taxonomic and phylogenetic assessments [1, 11, 13, 42]

The myoanatomy of tardigrades shows a clear heteronomous metamerism. In particular, the musculature associated with the ventral attachment sites and legs exhibits a metameric pattern. Specifically, the ventral musculature in all tardigrades investigated so far shows a repeating pattern centered around 5–7 ventromedian attachment sites in addition to a number of ventrolateral attachment sites. The comparison between ventromedian attachment sites, across Tardigrada, indicates that there has been a reduction in the number of these sites within several groups of the paraphyletic heterotardigrade order Arthrotardigrada (Fig. 5). Moreover, in many tardigrade species these originally paired sites appear to have fused during their evolutionary history. For example, in all investigated heterotardigrades as well as in *Milnesium tardigradum*, ventromedian attachment site *7* is unfused, indicating that the fused condition of this attachment site, as observed in eutardigrades, is most probably an apomorphic condition (Fig. 5). The reduction in the number as well as fusion of ventromedian attachments sites thus seems to represent a derived condition.

Five ventromedian attachment sites (sites *1*,*3*,*5*,*6*,*7*), all unfused, were recognized in *A. doryphorus*, a condition accompanied with relatively few ventral leg muscles. In addition, muscles connecting the ventral longitudinal muscle to ventromedian attachment sites seem to be lacking in this species. In marked contrast, in *E. sigismundi* several muscles connecting the ventral longitudinal musculature and the ventromedian attachment sites, such as muscles *1iii-1* and *5a* can be seen in Fig. 4a. Interestingly, it has been suggested that *B. pennaki*—another arthrotardigrade—in reality only has four true ventromedian attachment sites (sites *1*,*3*,*5*,*7*), as the positions of sites *2*, *4* and *6* are hard to discern [31]. *B. pennaki*, however, possesses a higher number of ventral muscles, as compared to *A. doryphorus*. As compared to the situation in *A. doryphorus* and *B. pennaki*, the 7 ventromedian attachment sites in *E. sigismundi* are arranged in a pattern that is more similar to that found in eutardigrades. Based on results from our comparative analyses, we propose that the presence of seven unfused ventromedian attachment sites is a condition that represents the ground pattern of Tardigrada. While many ventromedian attachment sites are fused in Eutardigrada, unfused sites are also commonly found among the members of this class. For example, in *Halobiotus crispae* (Halobiotidae) the fifth attachment site is unfused [35] and in another eutardigrade, *Richtersius coronifer* (Richtersiidae), both the third and the fifth attachment sites are unfused [25]. According to Schmidt-Rhaesa & Kulessa [30] *Milnesium tardigradum* seems to exhibit alternating unfused (*1*, *3*, *5* and *7*) and fused (*2*, *4*, and *6*) ventromedian attachment sites. These observations were more recently corroborated by Marchioro and co-workers [26], though these authors described the first ventromedian attachment site as fused in their analysis of *M. tardigradum* (see Fig. 3.B in [26]).

The relatively simple ventral trunk musculature of *A. doryphorus* includes a conspicuous thick ventral muscle composed of only four large sarcomeres that span the entire length of the trunk, attaching at three intermediate attachment points (*c,d,f*). Interestingly, in *A. doryphorus* the position of this ventral longitudinal muscle seems to have a more ventral position relative to the leg muscles than previously reported from other tardigrades. In the head region of *A. doryphorus* the ventral musculature is composed of a two-sarcomere inner and a three-sarcomere thick outer band that connect to the buccal musculature and to the clavae (Fig. 2a). Noticeably, phalloidin staining was found in the proximal part of all head appendages, both cirri and clavae, as well as in cirri of the trunk. In addition, *A. doryphorus* has a conspicuous and well-developed leg musculature and a delicate dorsal longitudinal musculature composed of two strands that extend into the head region.

Generally, echiniscoidean heterotardigrades, including *Echiniscoides sigismundi*, appear to have thicker, but also fewer muscles as compared to eutardigrades [current study; 26]. Within Echiniscoidea we found that the musculature of *E. sigismundi* is quite similar to that described for *Echiniscus testudo* [26]. However, it seems that *E. sigismundi* has only one thick ventral longitudinal muscle, whereas *E. testudo* has two that fuse at the attachment site *L* and remain so until terminating in attachment site *a* [26]. The ventromedian attachment sites and the ventral leg muscles of these two species, only differ slightly in selected structures. One of these differences is related to the third ventromedian attachment site, in which *E. sigismundi* differs from *E. testudo* by having a muscle, *3iii* (Fig. 4a), extending to intermediate attachment site *d* (equivalent to attachment site *L* in *E. testudo*), whereas *E. testudo* has a muscle extending to intermediate attachment site *K* (equivalent to attachment site *c* in *E. sigismundi*) [26]. Furthermore, the fourth leg pair also differs between the two species. In *E. testudo* the fourth leg pair receives one muscle from the dorsal musculature and one muscle from the lateral musculature [26]. However, in *E. sigismundi* the fourth leg pair receives four muscles from the dorsal musculature (Fig. 4, *w1-w4*) and the muscle from the lateral musculature connects with intermediate attachment site *g* (Fig. 4a, *8a*). Marchioro and colleagues [26] suggested that the thickness and reduction in number of muscles in *E. testudo* is related to the development of sclerotized cuticular plates. However, our study contradicts this hypothesis showing that the musculature of *E. sigismundi*, a species that lacks sclerotized cuticular plates, is as thick as that of *E. testudo*, if not thicker. This could thus be an indication that the condition of having thicker, fewer muscles is an apomorphy for echiniscoideans.

## Conclusions

Tardigrades have a complex myoanatomy that reflects the evolution of the phylum and its lineages as well as adaptations among specific species. In the current analysis we provide new myonatomical data on *Actinarctus doryphorus* (Tanarctidae) and *Echiniscoides sigismundi* (Echiniscoididae) highlighting features that characterize the two anatomically very distinct heterotardigrade species. Generally, the trunk musculature of *A. doryphorus* appears reduced and simplified as compared to other tardigrades, including other heterotardigrades. Our comparative analysis indicates that this reduction represents an apomorphic condition. In addition, cross-striation seems to be a feature common to the somatic muscles of heterotardigrades. A common feature to *E. sigismundi* and other echiniscoidean heterotardigrades seems to be the presence of relatively thick somatic muscle fibers. In our analyses we have focused on the ventral musculature of the trunk and we propose that 7 unfused ventromedian attachment sites are part of the ground pattern of tardigrades. The ventromedian attachment sites seem especially useful for comparative studies, as they seem to possess traits with value for phylogenetic analysis at several taxonomic levels. Specifically, the arrangement and structural pattern of these sites differs in particular details between classes, orders and families. In the future, a deeper understanding of the tardigrade myoanatomical ground pattern will require more investigations in order to include all major tardigrade lineages.

## Methods

### Collection and preservation of specimens

Specimens of adult *Actinarctus doryphorus ocellatus* Renuad-Mornant, 1971 (Fig. 1) used for CLSM and SEM were obtained from clean, coarse shell gravel collected off the coast of Roscoff, France [43], in a location known as Trezen ar Skoden between 2011 and 2013. The samples were taken with a Sanders dredge, at about 50 m water depth, through several transects with most specimens being retrieved from the following position: 48°45′563″N, 04°05′563″W to 48°45′560″N, 04°05′463″W. The sediment samples were soaked and stirred in freshwater, to cause the release of the meiofauna from the sediment grains by osmotic shock. The meiofauna was subsequently extracted by decantation through a 45 μm mesh net, transferred to Petri dishes with filtered seawater and sorted out using a stereomicroscope. For CLSM, specimens were fixed for 1 h at room temperature (RT) in 3–4% paraformaldehyde (PFA) in 0.1 M phosphate buffer solution (PBS; pH 7.4). Afterwards, all specimens were washed 3 × 15 min in PBS containing 0.1% sodium azide (NaN_3_) and stored at 4 °C. The specimens used for SEM were fixed for 1 h at RT in a 2.2% aldehyde fixative (1.2% PFA; 1% glutaraldehyde) in 0.05 M sodium cacodylate buffer containing 0.05 M sucrose (pH 7.4). The specimens were subsequently transferred to and stored at 5 °C in the sodium cacodylate buffer before being post fixed in 1% OsO_4_ in 0.1 M sodium cacodylate (pH 7.4) for 1 h at RT.

Specimens of *Echiniscoides sigismundi* Schultze, 1865 (Fig. 3) were sampled intertidally at Lynæs, Denmark (55°56.42′N, 11°51.15′E) in the period 2011–2013 by collecting barnacles of the species *Semibalanus balanoides* Linnaeus, 1767. The barnacles were gently broken apart, and subsequently freshwater shocked in order to expose the tardigrades living on their shells. *E. sigismundi* were located using a dissection microscope and relaxed in ddH_2_O for app. 30 min before fixation in 4% PFA in 0.1 M PBS (pH 7.4) for app. 1 h, at RT for CLSM or in 2.5% glutaraldehyde in 0.1 M sodium cacodylate buffer (pH 7.4) for 2 h for SEM.

### Phalloidin staining and confocal laser scanning microscopy

PFA-fixed specimens were washed 3 × 15 min in 0.1 M PBS and subsequently permeabilized overnight at 4 °C in 0.1 M PBS containing Triton X-100 (PBT, 5% for *Actinarctus doryphorus* and 1% for *Echiniscoides sigismundi*). The specimens were then stained in a 1:40 dilution of Alexa Fluor 488-conjugated phalloidin (Molecular Probes and Invitrogen) in PBT, for 24 h (*A doryphorus*) or 72 h (*E. sigismundi*) at 4 °C. The stained samples were washed in PBS (3 × 15 min) and mounted in Vectashield (Vector Laboratories Inc., Burlingame, CA, USA) or Fluoromount G (SouthernBiotech) antifade mounting medium on glass slides. A total number of 15 specimens of *A. doryphorus* and five specimens of *E. sigismundi* were investigated by CLSM. The fluorescence preparations were analyzed with a Leica DM 5000 CS microscope equipped with a Leica DM 5000 SP5 confocal laser scanning unit and a Leica TCS SP5 X microscope (Leica Microsystems, Wetzlar, Germany). Maximum projections of the image series were processed and edited with the 3D reconstruction software IMARIS (Bitplane AG, Zürich, Switzerland).

### Scanning electron microscopy

Fixed specimens for scanning electron microscopy were dehydrated through a graded series of ethanol, CO_2_-critical point dried (Autosamdri-815 critical point dryer, Tousimis Research Corporation, MD, USA) and coated with platinum-palladium (JEOL FC-2300 HR sputter coater, JEOL Ltd., Tokyo, Japan). Afterwards, tardigrades were examined and digitally photographed with a JEOL JSM-6335F field emission scanning electron microscope (JEOL Ltd., Tokyo, Japan).

## Supplementary information

**Additional file 1.** Movie of CLSM z-stacks used for the images shown in Fig. 2.

**Additional file 2.** Movie of CLSM z-stacks used for image shown in Fig. 4a.

**Additional file 3.** Movie of CLSM z-stacks used for image shown in Fig. 4b.

**Additional file 4.** Movie of CLSM z-stacks used for image shown in Fig. 4c.

## Data Availability

All data generated and analyzed during the current study are available from the corresponding author on reasonable request.

## Abbreviations

1–7: Ventromedian attachment site
a(a’)-f: Intermediate ventral attachment sites
ala: Wing-like extension of the cuticle
am: Anal muscles
an: Anus
A-W: Dorsal attachment sites (muscles associated dorsal sites are named with the corresponding lowercase letter followed by a number)
bm: Buccal muscles
B-W: Dorsal attachment sites
cE: Cirrus E
cg: Claw gland
ec: External cirrus/cirri
gm: Gonopore muscle
go: Gonopore
hm: Head muscles
ic: Internal cirri
lc: Lateral cirrus/cirri
mc: Median cirri
p3: Sensory organ of the third leg
p4: Sensory organ of the fourth leg
pb: Pharyngeal bulb
pc: Primary clavae
pil: Pillar
sc: Secondary clavae
T1-T3, τ0-τ3: Lateral attachment sites
